# *Solidago virgaurea* L. Plant Extract Targeted against *Candida albicans* to Reduce Oral Microbial Biomass: A Double Blind Randomized Trial on Healthy Adults

**DOI:** 10.3390/antibiotics9040137

**Published:** 2020-03-25

**Authors:** Isabelle Prêcheur, Yohan Rolland, Lilia Hasseine, François Orange, Adeline Morisot, Anne Landreau

**Affiliations:** 1 Université Nice Côte d’Azur, Faculté de Chirurgie Dentaire, Laboratoire Micoralis EA 7354, 06300 Nice, France; 2 Université Nice Côte d’Azur, Centre Hospitalier Universitaire, Pôle Odontologie, 06200 Nice, France; 3 Givaudan, Naturex, 84140 Avignon, France; yohan.rolland@givaudan.com; 4 Université Nice Côte d’Azur, Centre Hospitalier Universitaire, Laboratoire de Parasitologie-Mycologie, 06200 Nice, France; hasseine.l@chu-nice.fr (L.H.); landreau.a@chu-nice.fr (A.L.); 5 Université Nice Côte d’Azur, Faculté des Sciences, Centre Commun de Microscopie Appliquée (CCMA), 06000 Nice, France; francois.orange@univ-cotedazur.fr; 6 Université Nice Côte d’Azur, Centre Hospitalier Universitaire, Département de Santé Publique, 06200 Nice, France; morisot.a@chu-nice.fr; 7 Université Nice Côte d’Azur, Institut de Chimie de Nice CNRS UMR 7272, MBVB, 06000 Nice, France; 8 Université d’Angers, Faculté de Santé, 49045 Angers, France

**Keywords:** antiseptics, biodegradable, biofilm, *Candida albicans*, dental plaque, oral care products, mycobiome, *Solidago virgaurea*

## Abstract

Oral microbiome plays an important part on oral health and endogenous bacteria and fungi should not be eradicated. However, their proliferation must be controlled by oral hygiene care. In vitro, *Solidago virgaurea* ssp. *virgaurea* L. (SV) plant extract inhibits the adherence and hyphal formation of a fungus, *Candida albicans*. It reduces the biomass of *Candida*-bacterial biofilms but not fungal or bacterial growth. Unlike chemical antiseptics, like triclosan and chlorhexidine for instance, SV is a plant extract easily biodegradable. The purpose of this study was to assess the in vivo effectiveness of SV extract in reducing oral biomass. A randomized, double-blind clinical study, with dental plaque evaluation designed to assess the effectiveness of a fluorinated toothpaste containing SV (Bucovia™, Givaudan, Vernier, Switzerland) was conducted. Sixty-six subjects (SV group *n* = 33 vs. control *n* = 33) brushed their teeth twice a day for a 4-week period. Supragingival dental plaque was sampled. Total bacterial load (broad spectral bacterial quantitative Polymerase Chain Reaction (qPCR)), *C. albicans* and seven bacterial species were quantified by qPCR. In the Intervention group, there was a decrease of Total bacterial load (ΔD0D28 *p* = 0.005 and ΔD14D28 *p* = 0.026), *Streptococcus mutans* (ΔD0D14 *p* = 0.024) and *C. albicans* (ΔD0D28 *p* = 0.022). In the Control group Total bacterial load tended to decrease from baseline to day 28 (ΔD0D28 *p* = 0.062 and ΔD14D28 *p* = 0.009). Plaque Index and Gingival Index improved in both groups.

## 1. Introduction

Recent genomic and proteomic data revealed that more than 800 bacterial and 100 fungal phylotypes could colonize the oral cavity and contribute to oral biofilms. But most of the oral bacterial and fungal species cannot be grown in vitro [1,2,3]. This might be one reason why the role of fungi in oral health and disease has been underestimated [2,3,4]. *Candida albicans* is the prominent cultivable fungal species. But the oral mycobiome can harbor other *Candida* species, such as *Candida glabrata*, *Candida parapsilosis*, *Candida dublinensis*, *Candida krusei*, or *Candida tropicalis.* Prominent other oral fungi are *Aspergillus*, *Aureobasidium*, *Cladosporium*, *Epicoccum*, *Fusarium*, *Malassezia*, *Mucor*, *Penicillium*, *Saccharomyces*, *Trichophyton*, and *Wallemia* [5,6].

There is a dynamic balance between biofilms and host parameters, such as saliva, healthy diet, and oral hygiene. This balance limits microbial growth, and oral biofilms create a commensal protection against opportunistic pathogens [7,8,9]. Endogenous bacteria, but also *Candida* and other fungal species should not be eradicated or unbalanced in oral biofilms [2,3,4]. But any factor liable to unbalance oral ecosystems can lead to uncontrolled bacterial and fungal growth. As a result, there is a risk of oral infections, mainly dental caries, gingivitis, periodontitis, candidiasis, denture stomatitis, mucositis, delayed repair after oral surgery, and halitosis (bad breath) [10,11]. In healthy individuals carrying *C. albicans*, this fungus has a yeast form and colonization remains harmless [5]. But it can switch from yeast cells to virulent hyphal cells and cause oral candidiasis. *C. albicans* and Streptococcus species can co-increase their virulence in invasive candidiasis, but also in dental caries and in peri-implantitis [11,12].

Bacteria and fungi are adherent to oral surfaces and co-aggregating. They form clusters in biofilms, protected by a matrix which contains host and microbial components, including polysaccharides, glycoproteins, proteins, DNA, and lipids. Lipid constituents are poorly investigated. In oral biofilms, host components are derived from saliva, crevicular fluid and gingival epithelial cells. Bacteria and fungi embedded in the biofilm are more resistant to the innate immune system and to antimicrobial therapy [2,13]. In periodontal tissues, innate immunity is a semi-specific first line of defence, which initiates inflammatory reaction in response to oral microbiome dysbiosis [1]. Some microbial constituents trigger innate immunity, and other constituents and toxins are secondly recognized as microbial antigens by adaptative immunity. Innate immunity recruits immune cells to infection site (macrophages, mastocytes, endothelial cells, histiocytes, and fibroblasts) and from bloodstream (neutrophils, basophils, eosinophils, lymphocytes B and T, monocytes, plasmocytes, and platelets) [13,14]. During acute phase reaction, hepatocytes synthesize increased quantities of plasma proteins and glycoproteins. These plasmatic immunity mediators form complex activation systems (bradykinin system, fibrin/fibrinolysis system and complement system) [13,14].

A second group of immunity mediators are synthesized by various cell types. Main cell mediators are histamine, serotonin, eicosanoids, free radicals, cytokines, substance P, neurokinin, and enzymes involved in tissue destruction [13,14,15]. Bunte and Beikler recently reviewed periodontal immunity (2019) [16]. Briefly, the innate immune system activates phagocytosis, the complement system and the adaptative, immune system which is antigen-dependant and mediated by B and T cells. Humoral immunity fights pathogens via antigen-specific antibodies secreted in saliva and crevicular fluid, which neutralize microbial cells and their toxins. Humoral immunity also mediates allergy, autoimmunity, cell memory, and the production of cytokines. Cellular immunity involves macrophages, natural killer cells, apoptosis of bacteria-containing cells mediated by T cells, and secretion of cytokines by endothelial cells and fibroblasts [16]. As a result, microbial pathogenicity is controlled in healthy oral ecosystems.

In periodontitis, the response of the adaptative immune system is altered and the cytokine network is unbalanced, resulting in persistence of inflammation [14,16]. Typical inflammatory reaction is characterized by signs of pain, heat, redness, and swelling. However, destruction of periodontal tissues is most of time painless and (micro)-bleeding is a constant symptom. Periodontologists have paid a special attention to T cells, cytokine unbalance and free radicals [1,13,14,15,16]. T cells differentiate in several lineages of T helper (Th) cells. Th1 cells modulate cellular immunity, produce interleukin-2 (IL-2) and interferon gamma (IF-γ), and protect against intracellular bacteria, viruses and protozoa. Th2 cells modulate humoral immunity (activation of B cells and mastocytes), production of IL-4, IL-5, and IL-13, and protect against parasites. Th17 cells produce IL-17 and protect against extracellular bacteria and fungi at barrier sites. However, IL-17 could be an important proinflammatory mediator in periodontitis and immune-mediated inflammatory diseases [16]. According to Bunte and Beikler, modulation of the IL-17/IL-23 axis by monoclonal antibodies could have therapeutic applications [16]. Cytokines are proteins secreted by leukocytes and other cells during inflammatory stages. Main cytokines are Tumor Necrosis Factor—α (TNF-α), interleukins, chemokines and interferons. They are intercellular mediators. Periodontal disease is correlated with increased concentrations of serum IL-1, IL-6, prostaglandin and C-reactive proteins (CRP) [15]. Cytokines induce the production of endothelin-1 (ET-1) in endothelial cell, and ET-1 is secreted in serum and saliva. ET-1 constricts smooth muscular cells and stimulates proinflammatory cytokines in periodontal ligament cells [17]. In addition to early diagnosis and follow-up of periodontal lesions, modulation of ET-1 activity is another field of potential therapeutic applications [15]. Finally, phagocytosis of microorganisms by neutrophil leukocytes increases their oxygen consumption and release of oxygenated free radicals (superoxides, hydrogen peroxide, and hydroxide radical). There is an intensive research of drugs and natural compounds to prevent free radicals’ damages in inflamed tissues [18,19,20].

Most toothpastes and mouthwashes contain antiseptic agents, which are mainly bisbiguanides (chlorhexidine, hexetidine), metal ions (stannous fluoride), phenols (triclosan), and quaternary ammonium compounds (QACs: cetyl pyridinium chloride) [21,22,23,24,25]. In vivo, synthetic antiseptics are efficient in reducing oral biomass, but they can alter protective microbial biofilms and allow xerostomia, stomatitis or oral ulcer development [25]. Besides, some colonizing bacteria in the human microbiote possess resistance genes against QACs (*Qua*-genes such as *QuaC*). These resistance genes affect efflux pumps in bacteria and reduce bacterial susceptibility to other antiseptics, including cetrimide, benzalkonium chloride, and chlorhexidine [26,27]. In *Staphylococcus aureus* and *Enterococcus faecalis*, plasmid-borne *qac* genes can induce cross resistance with antibiotics such as fluoroquinolones [10,28]. In addition, the wide use of antiseptics in pharmaceuticals and personal care products generates their dispersal in the environment [29,30]. Either from food and/or topical antiseptic products, they are persistent in wastewater and pollute the environment. A long-term toxicity on aquatic animals and humans, as weak endocrine disruptors, is suspected [31,32,33,34,35].

As an alternative, some plants and plant extracts with antimicrobial, anti-inflammatory, and/or antioxidant properties are used in folk medicine to fight oral infections and especially gingivitis and periodontitis [36]. Plant extracts composition is complex, usually not fully elucidated. These extracts are by definition biodegradable in the environment, and in vitro and in vivo studies clarified some of their properties. Plant extracts with antimicrobial properties stricto sensu have been extensively investigated. Main natural compounds described as antimicrobial compounds are essential oils (complex mix of terpenoids and phenylpropane derivatives (aromatic compounds)) and tannins (phenolic products that can precipitate proteins, such as escin) [37,38,39,40,41,42]. Some vegetal compounds display anti-inflammatory properties, such as vitamins, mineral elements (iron), proteases (papain and bromelain, a mix of acid proteases), or some flavonoids (close to tannins, such as baicalin) [41,43,44,45,46]. Vitamins are essential organic substances that are required in small amounts to catalyze metabolic reactions [19]. Only vitamin C, vitamin D, and vitamin B3 could be useful for prevention and treatment of periodontal disease [18,47]. Vitamin C (ascorbate) is a water-soluble vitamin found in citrus fruits and many vegetables. It is the biologically active form of L-ascorbic acid. It is an antioxidant (a reducing agent) and coenzyme in several metabolic reactions, including hydroxylation of proline and lysine during collagen formation. This action on collagen structure is important to periodontium physiology and integrity. Vitamin C is also involved in inflammatory reaction, at the level of phagocytosis and wound healing [18]. Vitamin D is a liposoluble hormone found in the diet, mainly in fatty fish (cod liver oil), chocolate and whole milk. It is also synthesized in the skin from a derivative of cholesterol or ergosterol, under the action of ultraviolet B (UVB) radiation from the Sun. Vitamin D includes cholecalciferols and ergocalciferols, and regulates calcium in opposition to parathyroid hormone. Vitamin D facilitates the intestinal absorption of calcium and phosphorus, and the fixation of calcium on bones. Deficiencies could be involved in periodontitis [18]. Vitamin B3 or niacin is a water-soluble vitamin of the B complex, mainly found in food of animal origin (meat, fish) and in whole cereals. Vitamin B3 is required for the formation of coenzymes Nicotinamide Adenine Dinucleotide (NAD) and Nicotinamide Adenine Dinucleotide Phosphate (NADP) (energy metabolism). Vitamin B3 has vasodilating properties. Park et al. (2017) observed that in young adults, periodontitis was significantly associated with lower intakes of niacin, vitamin C, and iron, especially in women and current non-smokers [47]. Finally, iron is a metallic element, constituent of hemoglobins, cytochromes and iron-binding proteins. Iron is a micronutrient involved in the transport of oxygen and cellular redox reaction. Meat and meat products, as well as fish, are its best food sources. Many plants such as almonds, hazelnuts, oatmeal, spinach, lentils, and dates are also a source of iron, but in smaller quantities [47,48]. As a result, in addition to a balanced diet, multivitamins and antioxidants are currently consumed as dietary supplements [18,48,49,50].

In this context, a new natural component with original antimicrobial properties has been developed for oral care products (Bucovia™, Givaudan, Vernier, Switzerland). This plant extract was obtained from *Solidago virgaurea* ssp. *virgaurea* L. (SV), Asteraceae [51]. SV aqueous extracts mainly contains triterpene saponins, flavonoids and antocyanidines [52]. In vitro, SV extracts are not inhibitors of fungal or bacterial growth [51,52], but they inhibit *C. albicans* genes specific for adhesin and hyphal formation (*Hwp1*, *Als3*, *Ece1*, *Sap6*, and *Hgc1*) [53]. The resulting effects are an inhibition of yeast-hyphal transition and a reduced adherence to other *C. albicans* cells, to bacteria (*Streptococcus salivarius*) and to oral epithelial cells in culture (TR146 cell line). SV extracts also limit biofilm formation and reduce the biomass of pre-grown *Candida*-bacterial biofilms [53].

The global aim of this work was to avoid both synthetic chemical compounds and broad-spectrum antimicrobial effect in oral care products. The rationale of the present study was to develop a toothpaste with four specifications as follows: (i) It has to reduce oral biomass in order to maintain oral health but (ii) to preserve protective endogenous bacterial/fungal biofilms; (iii) to be harmless for man (no toxicity, absence of molecules suspected of weak endocrine disruptor properties) and (iv) to be harmless for the environment (do not destroy nor impoverish natural resources, not toxic for the environment and easily biodegradable). Specifications (iii) (chemical analysis) and (iv) were established in vitro. The null hypothesis was the assumption that any difference between patients’ groups was the result of chance (mainly *C. albicans* and Total bacterial load, Plaque Index and Gingival Index); that is, that the toothpaste had no effect. A randomized, double-blind clinical study involving 66 healthy adults confirmed that fluorinated toothpaste containing SV extract fulfilled specifications i, ii, and iii (no side-effects).

## 2. Results

### 2.1. Participants

#### 2.1.1. Flow Diagram

**Figure 1:**
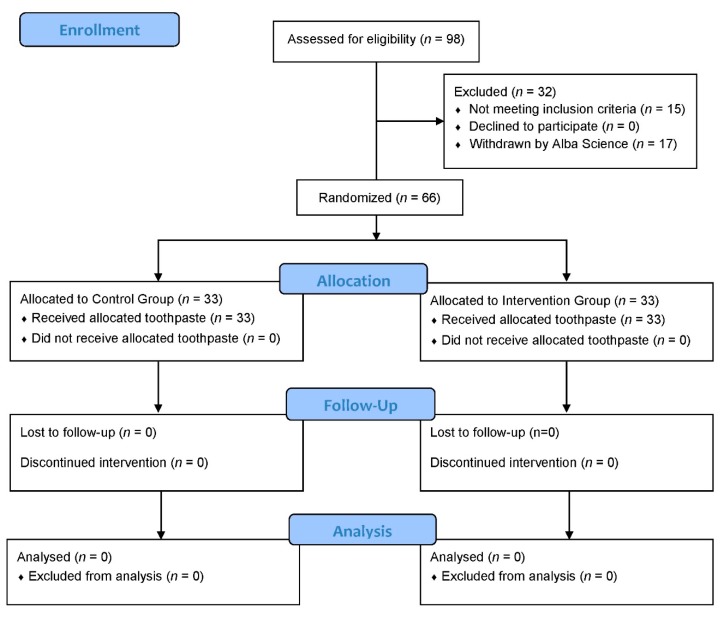
Flow diagram.

Alba Science clinical research associate pre-screened 98 subjects assessed for eligibility. Three participants were not fulfilling inclusion criteria, and 14 were included but did not wish to complete the study before the beginning of the study. Fifteen participants failed screening (exclusion criteria) and were withdrawn by Alba Science dental surgeon in charge to collect clinical data and microbial samples. Sixty-six participants completed the study (Figure 1). They brushed their teeth twice a day for a 4-week period.

No adverse events related to the product tested were reported.

#### 2.1.2. Baseline Characteristics of Participants

Sixty-six participants completed the study. Baseline characteristics of participants were as follows: 20 males (30.3%) and 40 females (69.7%); age 41.2 (sd = 11); Plaque Index 2.34 (sd = 0.31); Gingival Index 1.57 (sd = 0.25); Halitosis score 3.33 (sd = 0.98). Total bacterial load 1.6 × 10^9^ (sd = 6.1 × 10^8^); *C. albicans* (n = 13) 5.1 × 10^4^ (sd = 8.7 × 10^4^). Baseline demographic and clinical characteristics were similar (*p* > 0.05) in the Intervention group (33 subjects) vs. Control group (33 subjects) with one exception. There was a higher number of *Prevotella intermedia* carriers in the Intervention group vs. Control group at baseline (63.6% (21/33) vs. 24.2% (8/33); *p* = 0.002), but values became similar at D14 and D28. Subjects were not carrying all the bacterial and/or fungal species at baseline. The number of subjects carrying a given microbial species at baseline was as follows: *Aggregatibacter actinomycetemcomitans* 9.1% (6/66), *Fusobacterium nucleatum* 57.6% (38/66), *Porphyromonas gingivalis* (24.2% (16/66), *P. intermedia* 44.0% (29/66), *Treponema denticola* 95.5% (63/66), *Tannerella forsythia* 62.1% (41/66), *Streptococcus mutans* (97.0% (64/66), and *C. albicans* 20.0% (13/66).

### 2.2. Microbial Numeration

#### 2.2.1. Quantitative Polymerase Chain Reaction (qPCR) Results

Species-specific mean values were calculated for the subjects carrying the species at baseline (*n* = effective). qPCR numeration results are expressed as mean value (standard error) (Table 1). Paired Student tests (or paired Wilcoxon non parametric tests) were used to compare Intervention group vs. Control group. At baseline, the only difference between Intervention group and Control group was the number of subjects carrying *P intermedia*, which was higher in the Intervention group (21 vs. 8, *p* = 0.002). In the Intervention group, there was a decrease of *S. mutans* load from baseline to D14 (*p* = 0.024) and a decrease of *C. albicans* from baseline to the end of the study (*p* = 0.022) (specification i and ii).

#### 2.2.2. Total Bacterial Load

A linear mixed model was used to compare repeated measure (Total bacterial load) between visits at D0, D14 and D28 and between groups. Mixed analysis showed that Total bacterial load diminished from baseline to the end of the study in the Intervention group (ΔD0D28 *p* = 0.005 and ΔD14D28 *p* = 0.026) (specification i and ii). There was a tendency to decrease in the Control group from baseline to the end of the study (ΔD0D28 *p* = 0.062) and a decrease from D14 to D28 (ΔD14D28 *p* = 0.009) (Table 2).

### 2.3. Participants’s Scores and Questionnaire Answers

Sixty-six patients’ oral scores are detailed in Table 3. Briefly, Plaque Index, Gingival Index, and Halitosis score improved similarly, i.e., they diminished in both groups from baseline to the end of the study (paired Student or paired Wilcoxon tests).

Acceptability of the test toothpaste and control toothpaste was good (Table 4). However, there was a trend towards a longer clean feeling in the Intervention group vs Control group (Intervention group 269 ± 210 min vs. 193 ± 233 min), with approximately one-hour longer duration but without statistical significance (*p* = 0.260).

## 3. Discussion

This work describes a new and natural concept, with a demonstration of efficacy in man: “A SV extract in toothpaste reduced oral microbial biomass. Unlike synthetic antiseptics, this plant extract was able to reduce fungal and bacterial biomass in vivo, despite the fact that it was neither fungicidal nor bactericidal in vitro [51,52,53]. This result suggests that SV extract could be able to preserve protective oral biofilm [41]. In Table 1, comparison of species-specific microbial load from baseline (D0) to D14 and from baseline to D28 confirmed a synergy between *S. mutans* and *C. albicans* previously described by Falsetta et al. in 2014 [12]. The decrease in *S. mutans* biomass at D14 preceded the decrease in *C. albicans* biomass at D28. The chemical content of SV extract had been analyzed in a previous work and it did not contain molecules with known endocrine disruptor properties [53]. There was no antiseptic molecules stricto sensu in the formulation of the test toothpaste. In the present clinical study, no side-effects related to the test toothpaste were reported. This extract was obtained from a wild plant *S. virgaurea* L., which is not a rare or endangered species and it is easily biodegradable in the environment. These results fulfil the specifications (i) to (iv) which had triggered this work.

However, the first limitation of this work is that Carpegen Company did not transmit individual primer sequences and PCR conditions. Abusleme et al. (2013) previously determined Total bacterial load via real-time PCR using universal primers [54]. qPCR detection and quantification of periodontopathogenic bacteria, *S. mutans* and *C. albicans* have also been described by other authors, both in experimental in vitro biofilms and in vivo clinical samples [55,56,57,58,59]. However, in these conditions, the present work cannot be repeated by other authors independently of Carpegen Company. Besides, we did not used the ∆∆Ct method, because Carpegen did not transmit other data than the data listed in the Tables. Therefore, we were not able to calculate the relative fold gene expression of samples in qPCR assays.

Statistically significant reductions by the intervention were observed in Total bacterial load and *C. albicans* load. But interpretation of the bacterial load result is difficult to interpret, since the initial load in the control group was lower than the intervention group. Important individual variations were observed, as previously reviewed by several authors [1,2,4]. But in this work, microbial cell-numbers (genome equivalents) were very high in many cases. The lack of information about qPCR conditions limits interpretation of these quantitative results, but sampling protocol could be a significant parameter. In this study, rather large amounts of dental plaque were collected. According to inclusion criteria, subjects participating in the study had an average whole mouth dental plaque scores of >1.5 (Plaque Index) and an average gingival score of >1.0 (Gingival Index). The sampling method consisted in whole lower jaw scraping of supragingival dental plaque, similarly to Adams et al. sampling method. Usually, sulcus fluid samples for qPCR analyses are taken with paper points [60].

An exclusion criterion for the patients was “untreated caries or significant periodontal disease”. But qPCR data for the marker bacteria of caries (*S. mutans*) and periodontitis (Red complex species: *P. gingivalis*, *T. forsythia*, and *T. denticola*) were all very high (in the order of 10^6^) and thus suggestive of active diseases. The present results could be explained by sampling method and qPCR protocol. Shifts are observed between health and disease [1,60], and Edlund et al. (2018) observed in vitro that the most abundant taxa were not necessarily the most transcriptionally active taxa [61]. As regards oral streptococci, several authors demonstrated that *Streptococcus* was the dominant genus in supragingival dental plaque samples collected from healthy children and adults, either caries-free or displaying active caries lesions [2,3]. *S. mutans* was among moderate abundance species in dental plaque of children aged 5–7 [62], but *S. mutans* and *Lactobacillus salivarius* were prominent species in the dental plaque of individuals having dental caries [63]. In cavities *S. mutans*, could account for less than 1% of the total and active bacterial community [64]. There is a genomic variability of *S. mutans* strains, with large differences in resistance to low pH and oxidative stress, or in biofilm formation in the presence of sugar. Several authors pointed the risk of generalizing the properties of a given species [63,64]. As regards the Red complex species, Peterson et al. (2014) identified *T. forsythia* but not *P. gingivalis* or *T. denticola* in the dental plaque of children 52]. In adults, *P. gingivalis* and *T. denticola* share normal microbiota of healthy oral cavity, but poor oral hygiene enhances the count of *Veillonella*, *Prophyromonas*, *Fusobacterium*, and *Candida* [3]. Red complex species may be underrepresented in the subgingival plaque associated with periodontitis [65], but abundant in mature dental plaque [66].

In the present work, mature biofilm samples were analyzed. Analysis was based on qPCR identification and quantification of *C. albicans* and seven representative bacterial species. The impact of SV extract on *C. albicans* morphology and on fungal-bacterial co-aggregation was not investigated in vivo. It would be interesting to analyze by qPCR the expression profile of some clinical samples. For instance, *C. albicans* possesses important adhesion and hyphal growth associated genes (*Hwp1*, *Als1*, *Als3*, *Ece1*, and *Hgc1*). *S. mutans* has specific genes directly associated with extracellular polysaccharide matrix development (*Gtfb*, *Gftc*, *Gftd*, *Frua*, *Dexa*, and *Gbpb*), and with acid stress survival (*Atpd* and *Fabm*). *P. gingivalis* has major fimbrial adhesin (*Fima* and *Arca*) [63,67].

A second limitation of this work is that only 20% (13/66) of participants carried *C. albicans* at baseline. Ghannoum et al. (2010), for instance, identified *Candida* spp. in 75% of oral samples of 20 healthy adults, including *C. albicans* (40%), *Candida tropicalis* (15%), *Candida khmerensis* (5%), and *Candida metapsilosis* (5%) [5]. Mun et al. (2016), identified *Candida* spp. in 48% of 203 healthy adults, and of these *Candida* strains 85% were *C. albicans.* For these authors, *Candida* carriage was not associated to gender, age or presence of removable dentures, but associated to smoking and the presence of active carious lesions [68]. The low *C. albicans* carriage observed in the present study could result from exclusion criteria (untreated caries and smoking). Consequently, the scientific hypothesis based on unique inhibition of *C. albicans* adherence properties was not sufficient to explain the decrease in Total bacterial load that was observed with the group of subjects who used Bucovia™. Actually, saponins interfere with ergosterol, a molecule specific of fungal membranes [69]. Mycobiome analysis demonstrated that many fungal genera can colonize oral cavity, including non-cultivable genera and species [5,6,70,71].

Another explanation of the efficacy of *Solidago* extract in vivo could result from interactions between lipids, iron, and solidagosaponins, which have detergent and iron chelator properties. *Solidago* extracts have no hematologic toxicity in man, because saponins, which are present in several vegetables for instance, cannot cross the digestive barrier [72]. The resulting degradation of environmental nutriments could hamper microbial growth or adherence. In vitro, Yeast Extract Peptone broth and calf foetal serum are lipid-rich liquid media, which are routinely added to *C. albicans* cultures in order to promote growth and hyphal formation. In addition, oral anaerobic bacteria require iron or blood-containing culture media to grow. In vivo, gingivitis and periodontitis are microbial and inflammatory gum diseases characterized by occult or macroscopic bleeding. There is also a lipid- and iron-rich exudate called gingival fluid, issued from inflamed sulcus and periodontal pockets. This is why bacterial periodontopathogens, once settled, are auto-maintained [4]. Putative role of SV extract, as a detergent, against the environment of lipophilic oral *Malassezia* spp. is also addressed [70].

The third limitation of the present study was its duration limited to 4 weeks: Bucovia™ allowed a better microbial load reduction but Plaque Index or Gingival Index were similar. A mean additional freshness feeling of 76 min was observed, in parallel with the decrease of anaerobic bacteria (Table 1). *C. albicans* and total bacterial load reduction were observed after a 4-week period of *Solidago* toothpaste use. This is in line with a 2- to 3-week treatment duration, which is recommended for topical antifungals to treat oral candidiasis (nystatin, amphotericin B, miconazole, sertaconazole). This delay seems necessary to impact *Candida* in vivo and maybe other endogenous fungi. An additional clinical trial lasting 8 weeks or more, could confirm this hypothesis and improve clinical indices. For instance, recent clinical trials with toothpastes were conducted over periods ranging from 8 to 14 weeks [60,73,74].

This study was limited to 66 subjects and to a 4-week observation period. In the absence of significant Plaque Index and Gingival Index improvement, the results are mostly negative on a clinical point of view. However, the overall ecological effects on the plaque microbiome deserves attention. Indeed, Adams et al. (2017) tested a toothpaste containing enzymes and proteins in a double-blind, randomised, parallel group study involving 93 subjects [60]. The control was a fluorinated toothpaste. The test product (Zendium™, Unilever, Rotterdam, Nethelands) was the same fluorinated toothpaste containing “three enzyme systems (amyloglucosidase, glucose oxidase and lactoperoxidase), designed to promote the generation of hydrogen peroxide and hypothiocyanite, as well as three further protein components (lysozyme, lactoferrin and immunoglobulin IgG), designed to provide additional antimicrobial benefits”. After a 14-week period of use, the authors observed an increase in bacteria associated with gum health and a concomitant decrease in those associated with periodontal disease. *C. albicans* or other fungi, as well as clinical indexes such as Plaque Index or Gingival Index were not investigated. However, the authors considered that a toothpaste allowing a bacterial shift of the ecology of the oral microbiome was a susceptible to improve oral health [60].

Bucovia™ was free from tannins, known to induce an unpleasant astringent feeling in the mouth. However, we had difficulties to obtain a discolored extract adapted to oral care product formulations, because most of the filtered extracts and purified solidagosaponins had lost their anti-*Candida* efficacy. That means that in aqueous extracts, not only saponins but also other components of the plant, are active against biofilms. Indeed, Starks et al. identified some clerodane diterpens from *S. virgaurea* ethanol-ethyl acetate extracts, which had a moderate antibacterial activity and may provide a starting point for the synthesis of more active compounds [75]. Another study found that methanol and water extracts of *S. virgaurea* showed antioxidant activity and try to demonstrate a connection between antioxidant and antimicrobial activity [76]. Due to the chemical complexity of plant extracts, the exact role of major plant components or synergies between plant components still remains in question. However, several clinical studies demonstrated the efficacy of various herbal extracts to reduce plaque deposits and to improve oral conditions such as chronic gingivitis and post-operative dental surgery [39,40,41,44]. The review of literature revealed that vitamins and antioxidants of vegetal origin could interfere with inflammatory reaction, free radicals and endothelin in damaged periodontal tissues [17,18,19,48]. They could also interfere with microbial growth and it would be interesting to investigate synergies with *S. virgaurea* extract, eventually as topical adjuncts. In order to confirm the efficiency of this herbal extract, it would be interesting to assay the host inflammatory response on a long-term survey [13]. Saliva and sulcular levels of inflammatory biomarkers associated with chronic periodontitis could be investigated, such as free radicals, cytokines, matrix metalloproteinase (MMP)-2 and -8, tissue inhibitor of metalloproteinases (TIMP)-2 complex, or endothelin-1, for instance [15,17,50]. A synergy between SV extract and dietary supplements, in particular vitamins with antioxidant properties could be investigated too [20,50].

Laboratory analyses confirmed that Bucovia™ was easily biodegradable (vegetal origin) and that experimental toothpaste formulated at 0.3% final concentration was not toxic on mammals. In comparison, some compounds present in essential oils used in toothpastes, such as thymol or carvacrol, are also obtained from plants and they are easily biodegradable too. However, unlike Bucovia™ they have broad-spectrum bactericidal and fungicidal properties, which have been demonstrated in vitro [37]. Using SV extract we aimed to reduce oral biomass in order to maintain oral health, but we also wanted to avoid a broad-spectrum action, in order to preserve protective endogenous bacterial/fungal biofilms. Vegetal proteases such as papain or bromelain could also help to disintegrate oral biofilm in a non-specific manner [41,44,46].

## 4. Materials and Methods

### 4.1. Experimental Toothpastes

#### 4.1.1. Plant Material

*Solidago virgaurea* ssp. *virgaurea* L. is a perennial plant common in Europe at low altitude (below 1500 m). It grows mainly on fallow lands (not cut meadows). SV is recognizable by its elongated and branched golden yellow flower heads that bloom in summer. It was harvested in its wild state in Poland, by trained harvesters that are able to recognize the species (Naturex for Givaudan, Poland Division). *S. virgaurea* has been used in European folk medicine for centuries, mostly in traditional use as herbal tea with diuretic properties [72]. *S. virgaurea* has been thoroughly investigated and it is known to contain flavonoids, triterpenes glycosides and triterpenoids saponins. Plant authentication was achieved with thin layer chromatography (TLC) analysis performed on the raw material to ensure botanical identification and to detect conform vs. non-conform batches [52].

#### 4.1.2. *Solidago virgaurea* Extraction Process

Three kilograms of air-dried *S. virgaurea* were obtained from 8 kg of aerial parts of fresh plant. Ground dry herb (500 g) was first extracted with a water/ethanol mixture (70/30) at 50 °C. After separation of the charged liquid and the extracted herb, the liquid was filtered and the ethanol was removed by vacuum distillation. A fractionation step was performed by precipitation of undesirable compounds with addition of ethanol on the concentrate (ratio 1:10). Ethanol was removed by vacuum distillation, and the final concentrate was formulated with vegetal propylene glycol, a suitable solvent to solubilize all active compounds. *S. virgaurea* extract (Bucovia™) displayed the following physical and chemical characteristics: Liquid, light amber to amber, water soluble, specific gravity (20 °C): 1.050–1.015, titrated in solidagosaponins and flavonol o-glycosides.

**Figure 2:**
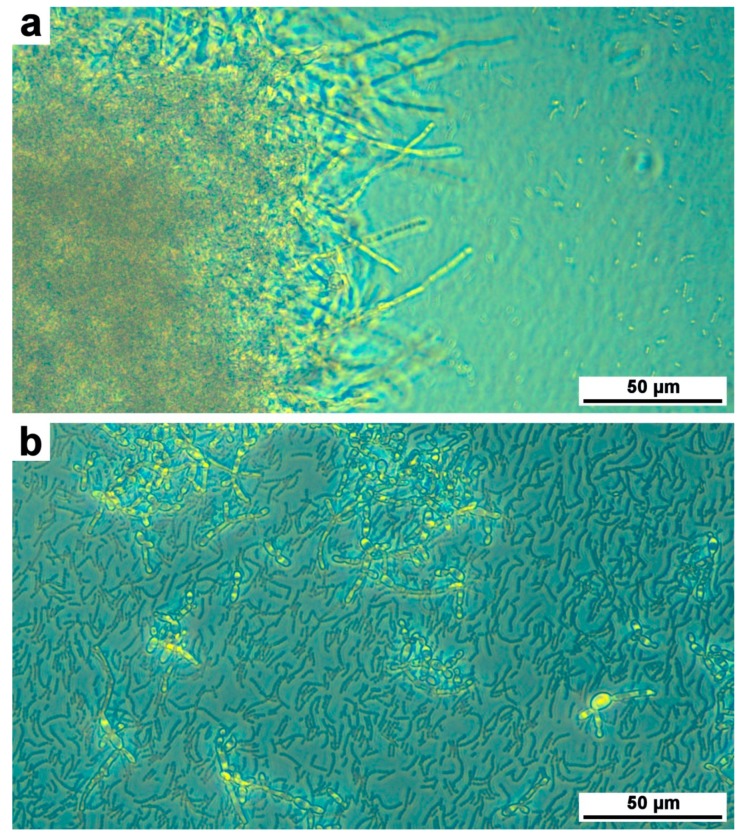
Co-culture of *Candida albicans* and *Streptococcus salivarius*. (**a**) Without Bucovia™. (**b**) With Bucovia™ 0.3% (×32).

**Figure 3:**
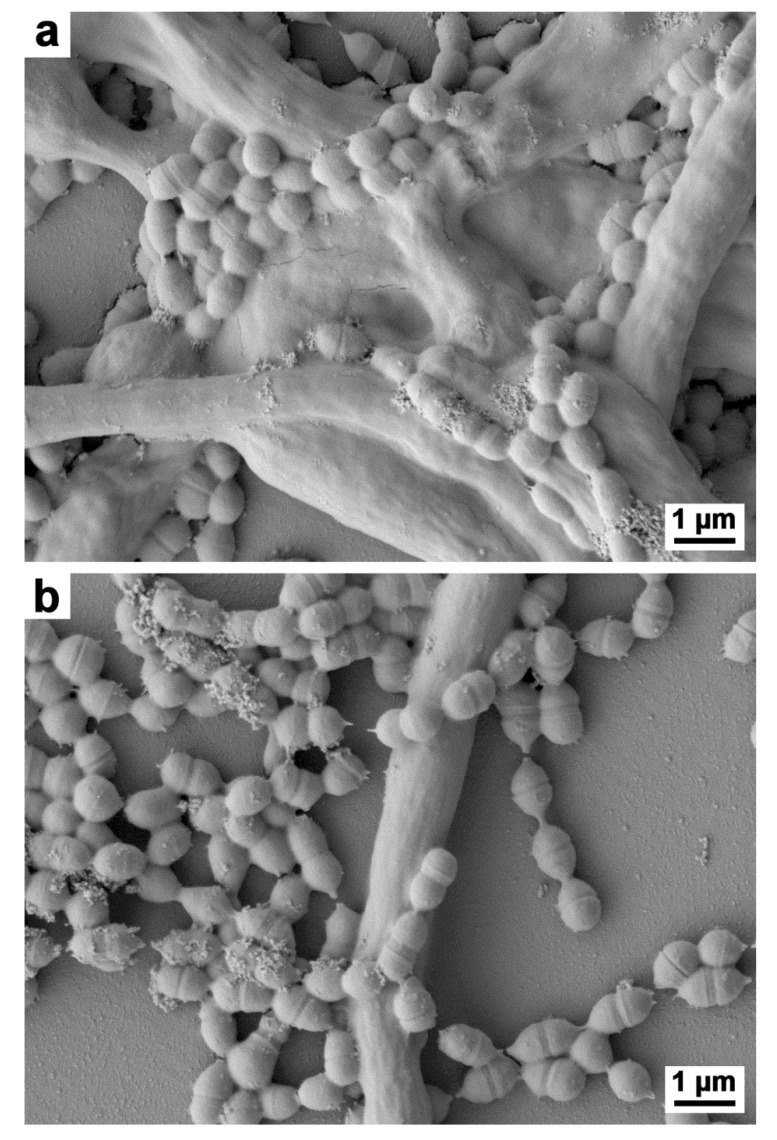
Co-culture of *Candida albicans* and *Streptococcus salivarius*. (**a**) Without Bucovia™. (**b**) With Bucovia™ 0.3% (×10,000).

In order to validate SV extract properties before the production of experimental toothpaste, we used oral fungal–bacterial biofilm models in vitro, as previously described [77]. Microscopic examination showed anti-adherence properties of Bucovia™ 0.3%, tested in vitro against co-culture of *C. albicans* and *S. salivarius* [51,53]. Phase contrast microscopy evidenced dispersion of *Candida* and *Streptococcus* cells in culture medium, and short and altered hyphal forms (Figure 2). Scanning electron microscopy evidenced a strong reduction of bacterial cells adherence to *Candida* cells (Figure 3).

#### 4.1.3. Toothpastes Formulation and Safety Controls

The product tested was fluorinated toothpaste with Bucovia™ 0.3% final vs. control toothpaste with the active replaced by distilled water. Test and control toothpastes contained sodium fluoride at 0.15%. Excipients were saccharin, sorbitol, glycerine, xanthan gum, carrageenan, hydrated silica, sodium lauryl sulfate, titanium dioxide, and aroma (doublemint flavour); pH 6.6 ± 0.2. Regulatory safety controls of the experimental toothpaste for the clinical study were carried out by an external independent toxicologist (Evic France laboratory, Vincennes, France). In foreseeable conditions of exposure in cosmetic products, no significant concern was associated to Bucovia™, which contained SV (aerial parts) extract and natural propylene glycol. Biodegradability fulfilled the standardized Test number 301 of the Organisation for Eonomic Co-operation and Development (OECD 301 B). Bucovia™ was reported to be easily biodegradable (>75% in 28 days). It was also compliant with all the applicable requirement of the Registration, Evaluation, Authorisation, and Restriction of Chemicals (REACH) Regulation of European Commission (EC) N° 1907/2006 and its modifications and updates. These are mandatory and regulatory tests for authorization to use a cosmetic product in a clinical study. European regulations require that these tests be carried out by laboratories approved by the Ministry of Health [78]

### 4.2. Clinical Study

#### 4.2.1. Hypothesis, Protocol Design, and Ethics Committee

We hypothesized that fluorinated toothpaste containing Bucovia™ could reduce oral microbial load by inhibiting *C. albicans* adherence and the resulting fungal-bacterial development in vivo. We realized a clinical study involving 66 healthy volunteers, Intervention group vs. Control group (33 vs. 33), who used experimental toothpastes twice a day over 28 days. It was a randomized, double-blind evaluation of plaque reduction. The study was achieved by Alba Science (Edinburgh, UK). The protocol was authorized by Reading Independent Ethics Committee (study n° 212415, Woodley, UK).

#### 4.2.2. Endpoints

- Primary endpoint

Primary endpoint was microbial load analysis pre, per and post-treatment, recorded at baseline (D0), after 2 weeks (D14), and after 4 weeks (D28). After 12 h without oral hygiene, supragingival plaque was harvested by a dental surgeon and pooled. Samples were collected and then stored, shipped within 2 days and kept at −20 °C until analysis. *C. albicans* and seven bacterial species were identified and quantified by qPCR: *Aggregatibacter actinomycetemcomitans*, *Fusobacterium nucleatum*, *Porphyromonas gingivalis*, *Prevotella intermedia*, *Treponema denticola*, *Tannerella forsythia*, and *S. mutans*. Microbial analyses were achieved by a molecular diagnosis company (Carpegen GmbH, Münster, Germany).

- Secondary endpoints

Secondary endpoints were oral scores recorded at D0, D7, D14, D21, and D28: Plaque Index, Gingival Index and Halitosis score [79]. Patients’ questionnaire responses were recorded at the end of the study (D28): Dry mouth improvement, longer freshness feeling, irritated gum soothing, gum redness and swelling reduction, bleeding of gum or reduction of plaque formation on teeth, and responses on product use. Adverse events were recorded throughout the use period. Restriction during the study was no use of antiseptic mouthwash or other anti-plaque toothpastes containing an active agent such as triclosan.

#### 4.2.3. Inclusion and Exclusion Criteria

- Inclusion criteria

Inclusion criteria were as follows: >18 in good general health; males and females; having at least 20 natural teeth including 5 teeth (excluding 3rd molars) in each quadrant; average whole mouth dental plaque scores of >1.5 by the Turesky modification of the Quigley-Hein index (Plaque Index) and with average score of >1.0 by the Loe-Silness Index (Gingival Index) [79].

- Exclusion criteria

Exclusion criteria were: Untreated caries or significant periodontal disease; full or partial dentures wearers; current orthodontic treatment; oral piercings; smokers or those who have smoked in the preceding five years; and taking dietary supplements (e.g., multivitamins, antioxidants, fish oils). No use of antiseptic mouthwash or toothpaste containing an anti-plaque active agent during the previous month was mandatory. Every subject signed informed consent.

#### 4.2.4. Randomization

Group allocation was randomized by an Alba Science clinical research associate in two blocks with an ALEA function, Excel, and Microsoft Word 2010 suite of programs. Each toothpaste tube was attributed a random number (test and control tubes). An Alba Science dental surgeon investigator enrolled participants and assigned participants to intervention by order of inclusion. Participants were dispensed toothpaste and new standard toothbrush along with diary and instructions of use. The dental surgeon, participants, and later people assessing outcomes were blind to treatment allocation.

#### 4.2.5. Dental Plaque Sampling

A single and trained dental-surgeon was in charge of all of the patients participating in the study. Supragingival dental plaque was scraped from the lower teeth, spread onto five paper picks and stored in a labelled Eppendorf tube. Carpegen sent the materials for transportation. No medium was used and Eppendorf tubes were immediately frozen at −20 °C. Eppendorf tubes were sent from Alba Science (Edinburgh, UK) to Carpegen (Muenster, Germany) in a carboglace, by express mail.

#### 4.2.6. qPCR Analysis

Microbial analyses were carried out by Carpegen GmbH. Total bacterial load was measured by a broad spectral “universal” qPCR, according to Carpegen protocol. The samples were analyzed by hydrolysis probe-based real-time PCR assays using the real-time PCR instrument LightCycler 480 II (Roche Molecular Diagnosis, Pleasanton, CA, USA). Singleplex PCR reactions were used, including negative and positive controls for each assay, as well as an internal control reaction to exclude inhibitory effects. Quantification was performed by analyzing serial dilutions of plasmid controls containing the respective cloned target amplicon. Further details of qPCR analysis protocol, including primer and probe sequences, were Carpegen proprietary and could not be disclosed to authors.

#### 4.2.7. Statistical Method

Data are reported as mean (standard deviation) or number (%). Patient’s characteristics were compared, Intervention group vs. Control group, using Student’s *t* test or Fisher’s exact tests for categorical variables and Chi-square test or Wilcoxon–Mann–Whitney test for continuous variables. Microbial load between baseline, 2-weeks, and 4-weeks and between each groups were compared using paired tests (Wilcoxon–Mann–Whitney or *t*-test) in a linear mixed model. Differences were considered as statistically significant for *p* < 0.05. Statistical analyses were performed using R version 3.5.1. Statistical analyst was blind to intervention.

## 5. Conclusions

In conclusion, inhibition of *C. albicans* adherence properties, and maybe inhibition of adherence/hyphal formation of other oral fungi, was an original approach for reducing oral biomass because they prevent the development of co-aggregating bacteria. The impact on bacterial microbiota in vitro and in vivo needs further works based on imagery, genomics and proteomics. Additional work could confirm whether this hypothesis could be extended to other hyphae-forming fungal genera in oral and dermatological mycobiome. The clinical trial allowed us to demonstrate that fluorinated toothpaste containing *Solidago* extract was efficient and safe for reducing oral biomass. Unlike antiseptics, such as triclosan or chlorhexidine, plant extract of *S. virgaurea* is both biodegradable and harmless for host and environment. When they are carefully validated in vitro and in vivo, some plant extracts alone or in combination seem to be a valuable alternative to synthetic antiseptics, at least for daily oral hygiene.

## Figures and Tables

**Table 1:** Microbial numeration at baseline (D0), D14, and D28.

Microbial Load	D0	D14	D28	ΔD0D14	ΔD0D28
(SE ^1^)				*p*-Value	*p*-Value
**Intervention group (*n*^2^)**					
Total bacterial load	1.7 × 10^9^	1.6 × 10^9^	1.2 × 10^9^	0.996	0.005
	(6.2 × 10^8^)	(6.3 × 10^8^)	(5.9 × 10^8^)		
*A. a*^3^ (4)	9.9 × 10^4^	1.4 × 10^4^	5.3 × 10^4^	0.099	0.100
	(8.1 × 10^4^)	(1.7 × 10^4^)	(8.5 × 10^4^)		
*F. nucleatum* (21)	1.5 × 10^6^	1.5 × 10^6^	1.0 × 10^6^	0.509	0.370
	(2.6 × 10^6^)	(2.3 × 10^6^)	(1.7 × 10^6^)		
*P. gingivalis* (8)	1.2 × 10^7^	1.0 × 10^7^	9.6 × 10^6^	0.624	0.262
	(1.7 × 10^7^)	(1.9 × 10^7^)	(1.4 × 10^7^)		
*P. intermedia* (21)	3.7 × 10^6^	3.8 × 10^6^	5.2 × 10^6^	1	0.651
	(4.5 × 10^6^)	(5.0 × 10^6^)	(8.6 × 10^6^)		
*T. denticola* (32)	6.7 × 10^5^	3.2 × 10^6^	2.9 × 10^6^	0.633	0.743
	(4.8 × 10^6^)	(4.0 × 10^6^)	(4.2 × 10^6^)		
*T. forsythia* (20)	2.2 × 10^6^	1.4 × 10^6^	2.1 × 10^6^	0.145	0.232
	(3.8 × 10^6^)	(3.5 × 10^6^)	(4.4 × 10^6^)		
*S. mutans* (32)	4.3 × 10^6^	2.6 × 10^6^	2.9 × 10^6^	0.024	0.090
	(8.0 × 10^6^)	(5.9 × 10^6^)	(8.8 × 10^6^)		
*C. albicans* (7)	6.8 × 10^4^	8.9 × 10^3^	1.3 × 10^3^	0.446	0.022
	(1.1 × 10^5^)	(1.0 × 10^4^)	(2.6 × 10^3^)		
**Control group (*n*)**					
Total bacterial load	1.5 × 10^9^	1.6 × 10^9^	1.1 × 10^9^	0.987	0.062
	(6.0 × 10^8^)	(5.3 × 10^8^)	(6.8 × 10^8^)		
*A. a*^3^ (2)	2.2 × 10^5^	5.6 × 10^4^	7.5 × 10^5^		
	(3.0 × 10^5^)	(7.5 × 10^4^)	(1.0 × 10^6^)	-	NA ^4^
*F. nucleatum* (17)	2.0 × 10^6^	2.4 × 10^6^	2.4 × 10^6^	0.129	0.754
	(4.6 × 10^6^)	(4.8 × 10^6^)	(4.3 × 10^6^)		
*P. gingivalis* (8)	4.8 × 10^6^	4.7 × 10^6^	6.8 × 10^6^	0.944	0.362
	(6.5 × 10^6^)	(1.1 × 10^7^)	(9.3 × 10^6^)		
*P. intermedia* (8)	6.8 × 10^6^	6.9 × 10^6^	8.6 × 10^6^	0.293	0.441
	(7.1 × 10^6^)	(8.6 × 10^6^)	(9.5 × 10^6^)		
*T. denticola* (31)	2.8 × 10^6^	2.1 × 10^6^	2.9 × 10^6^	0.259	0.906
	(3.8 ×10^6^)	(2.3 × 10^6^)	(4.6 × 10^6^)		
*T. forsythia* (21)	1.1 × 10^6^	8.0 × 10^5^	1.9 × 10^6^	0.130	0.651
	(1.9 × 10^6^)	(1.5 × 10^6^)	(3.9 × 10^6^)		
*S. mutans* (32)	7.4 × 10^6^	7.2 × 10^7^	4.9 × 10^6^	1	0.326
	(1.3 × 10^7^)	(1.3 × 10^7^)	(1.3 × 10^7^)		
*C. albicans* (6)	3.1 × 10^4^	5.9 × 10^4^	1.4 × 10^4^	0.674	0.833
	(4.9 × 10^4^)	(6.7 × 10^4^)	(1.3 × 10^6^)		

^1^ SE: standard error; ^2^ *n* = number of subjects carrying a given microbial species; ^3^ *A. a: Aggregatibacter actinomycetemcomitans*; ^4^ NA: not applicable because only two observations.

**Table 2:** Comparison of Total bacterial load in Intervention group vs. Control group.

Total Bacterial Load	Difference	CI ^1^-Lower	CI ^1^-Higher	*p*-Value
**Intergroup comparison**				
Intervention D0 vs. Control D0	−1.7 × 10^8^	−6.1 × 10^8^	2.7 × 10^8^	0.869
Intervention D14 vs. Control D14	−1.5 × 10^7^	−4.5 × 10^8^	4.2 × 10^8^	0.999
Intervention D28 vs. Control D28	−6.2 × 10^7^	−4.9 × 10^8^	3.7 × 10^8^	0.998
**Intragroup comparison**				
Intervention D0 vs. Intervention D14	6.7 × 10^7^	−3.3 × 10^8^	4.7 × 10^8^	0.996
Intervention D0 vs. Intervention D28	4.9 × 10^8^	9.8 × 10^7^	9 × 10^8^	0.005
Intervention D14 vs. Intervention D28	4.3 × 10^8^	3.1 × 10^7^	8.3 × 10^8^	0.026
Control D0 vs. Control D14	−8.9 × 10^7^	−4.9 × 10^8^	3.1 × 10^8^	0.987
Control D0 vs. Control D28	3.9 × 10^8^	−1.1 × 10^7^	7.9 × 10^8^	0.062
Control D14 vs. Control D28	4.8 × 10^8^	7.8 × 10^7^	8.8 × 10^8^	0.009

^1^ CI: Confidence interval.

**Table 3:** Comparison of oral clinical parameters from baseline (D0) to the end of the study (D28).

Mean Value (SE ^1^)	D0	D28	p
**Intervention group (*n* = 33)**			
Plaque Index	2.4 (0.4)	2 (0.6)	<0.001
Gingival Index	1.6 (0.3)	1.1 (0.6)	<0.001
Halitosis score	3.5 (0.8)	2 (1.2)	<0.001
**Control group (*n* = 33)**			
Plaque Index	2.3 (0.3)	1.8 (0.5)	<0.001
Gingival Index	1.5 (0.2)	0.9 (0.6)	<0.001
Halitosis score	3.1 (1.1)	1.5 (0.9)	<0.001

^1^ SE: Standard error.

**Table 4:** Questionnaire responses about the test toothpaste and control toothpaste at the end of the study.

Questionnaire Responses at D28		Yes		No	
No.	Question	Control group	Intervention group	Control group	Intervention group
1	The tested product improves dry mouth?	8	7	25	26
2	The tested product gives clean feeling in mouth?	23	22	10	11
3	If the tested product gives clean feeling in mouth, how long? ^1^	NA ^2^	NA ^2^	NA ^2^	NA^2^
4	The tested product delays bad breath occurring?	20	15	13	18
5	The tested product soothes irritated gum?	11	9	22	24
6	The tested product reduces gum redness and swelling?	11	12	22	21
7	The tested product reduces gum bleeding?	14	11	19	22
8	The tested product reduces biofilm formation (plaque) on teeth?	20	18	13	15

^1^ Question 3: Control group 69.7% answers (23/33): 193 ± 233 min (1.5 min to all day (12 h)); Intervention group 66.7% answers (22/33): 269 ± 210 min (5 min to all day (12 h)). ^2^ NA: not applicable.
